# Arabinoxylans-Based Oral Insulin Delivery System Targeting the Colon: Simulation in a Human Intestinal Microbial Ecosystem and Evaluation in Diabetic Rats

**DOI:** 10.3390/ph15091062

**Published:** 2022-08-26

**Authors:** Ana L. Martínez-López, Elizabeth Carvajal-Millan, Rafael Canett-Romero, Satya Prakash, Agustín Rascón-Chu, Yolanda L. López-Franco, Jaime Lizardi-Mendoza, Valerie Micard

**Affiliations:** 1 Research Center for Food and Development, CIAD, A.C. Carretera a La Victoria Km. 0.6, Hermosillo 83304, Sonora, Mexico; 2 NANO-VAC Research Group, Department of Chemistry and Pharmaceutical Technology, University of Navarra, 31008 Pamplona, Spain; 3 Departamento de Investigación y Posgrado en Alimentos, Universidad de Sonora, Rosales y Blvd. Luis D. Colosio, Hermosillo 83000, Sonora, Mexico; 4 Biomedical Technology and Cell Therapy Research Laboratory, Department of Biomedical Engineering, Faculty of Medicine, Artificial Cell and Organs Research Centre, McGill University, Montreal, QC H3A 0G4, Canada; 5 Montpellier SupAgro-INRA-UM-CIRAD, JRU IATE, 2, Place Pierre Viala, CEDEX 01, 34060 Montpellier, France

**Keywords:** insulin, oral drug delivery, electrospray, bioavailability, colon, SHIME

## Abstract

Arabinoxylans (AX) microcapsules loaded with insulin were prepared by enzymatic gelation of AX, using a triaxial electrospray method. The microcapsules presented a spherical shape, with an average size of 250 µm. The behavior of AX microcapsules was evaluated using a simulator of the human intestinal microbial ecosystem. AX microcapsules were mainly (70%) degraded in the ascending colon. The fermentation was completed in the descending colon, increasing the production of acetic, propionic, and butyric acids. In the three regions of the colon, the fermentation of AX microcapsules significantly increased populations of *Bifidobacterium* and *Lactobacillus* and decreased the population of *Enterobacteriaceae*. In addition, the results found in this in vitro model showed that the AX microcapsules could resist the simulated conditions of the upper gastrointestinal system and be a carrier for insulin delivery to the colon. The pharmacological activity of insulin-loaded AX microcapsules was evaluated after oral delivery in diabetic rats. AX microcapsules lowered the serum glucose levels in diabetic rats by 75%, with insulin doses of 25 and 50 IU/kg. The hypoglycemic effect and the insulin levels remained for more than 48 h. Oral relative bioavailability was 13 and 8.7% for the 25 and 50 IU/kg doses, respectively. These results indicate that AX microcapsules are a promising microbiota-activated system for oral insulin delivery in the colon.

## 1. Introduction

In recent decades, several studies have been focused on the design of non-invasive alternative routes for insulin delivery. Oral is the most effective, safe, and convenient route for the non-invasive administration of insulin [1]. However, successful oral delivery of insulin involves overcoming the barrier of enzymatic degradation and achieving epithelial permeability [2]. Therefore, recent approaches have been dedicated to protecting insulin from degradation using encapsulation devices, allowing a targeted delivery to specific sites of absorption, such as the colon. Colon-specific drug delivery presents several advantages, including limited proteolytic enzymes, a prolonged residence time, higher tissue responsiveness to absorption enhancers, and natural absorption [3]. The degradation of non-starch polysaccharides by colonic microflora is, among others, a foremost triggering mechanism to attain colon-specific drug delivery. This strategy is based on the high diversity of bacteria present in the colon and their capacity to produce a broad spectrum of enzymes responsible for the degradation of polysaccharides [4]. In this regard, the most active enzymes include β-D-galactosidase, β-D-fucosidase β-D-glucosidase, α-L-arabinofuranosidase, and β-xylosidase. The latter two are associated with the direct degradation of arabinoxylans [5]. Arabinoxylans (AX) are non-starch polysaccharides with high potential as prebiotics, and their consumption has been related to the treatment and prevention of obesity, type II diabetes, and colorectal cancer. These beneficial health effects are associated with their ability to modulate gut microbiota [6]. Moreover, the AX can form covalent gels via enzymatic cross-linking of ferulic acid (FA) esterified to the molecule [7,8,9]. AX gels are receiving increasing attention as oral delivery systems for biomolecules with therapeutic purposes. The AX resists the attack of digestive enzymes in the upper gastrointestinal (G.I.) system and could protect entrapped biomolecules until they reach the colon. In fact, previous studies revealed that the biodegradability of the AX network by colon microbiota is modulated by changes in their structural parameters, mainly the cross-link density and mesh size [10]. Insulin entrapment within AX microspheres has been recently reported [11,12]. The authors found that the formulation process via enzymatic oxidation did not affect the biological activity of insulin. However, the mechanism by which it performs the release of insulin has not yet been explored. Furthermore, the microencapsulation of insulin in the core shell offers further advantages than the entrapment of microspheres [13]. Therefore, in this work, we used an electrospray system mounted with a triaxial nozzle to generate insulin-loaded AX microcapsules. The behavior of these microcapsules under physiologically appropriate conditions was evaluated using a dynamic computer-controlled human simulated G.I. model. This in vitro model is a scientifically validated simulator of the physiological, enzymatic, and microbiological conditions of the human digestive system, which has been already used in several studies to evaluate the effect of prebiotics and probiotics in the gut [14,15,16]. Finally, the main objective of the present study was to determine whether insulin encapsulated in AX microcapsules induced a hypoglycemic effect and improved insulin bioavailability after oral administration to diabetic rats.

## 2. Results and Discussion

### 2.1. Characterization of Insulin-Loaded AX Microcapsules

**Figure 1:**
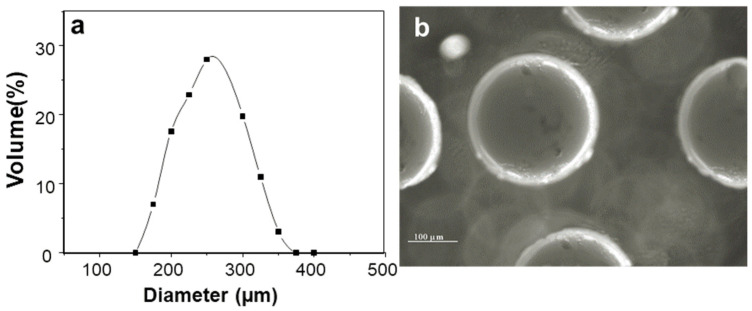
Characterization of insulin-loaded AX microcapsules: (**a**) diameter distribution; (**b**) contrast optical microscope observation.

The AX microcapsules presented a size distribution from 176 µm up to 398 µm, with an average diameter of 250 µm (Figure 1a). These particle size values were higher than those reported for poly-lactic microcapsules prepared with the electrospray method (<10 µm) [17,18], but close to those reported for chitosan/alginate microcapsules (150 µm) [19]. The insulin loading was calculated to be about 83 µg/mg microsphere, with an encapsulation efficiency of 85%. Optical micrographs of AX microspheres showed a spherical shape and no aggregation of particles (Figure 1b).

### 2.2. Morphology Changes of Insulin-Loaded AX Microcapsules in the Simulated Human G.I. Environment

**Figure 2:**
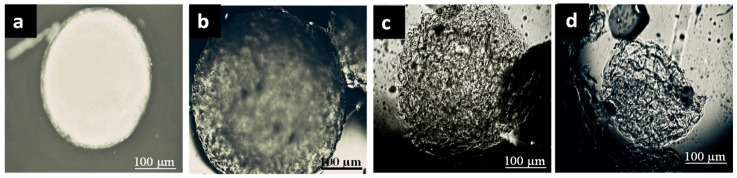
Optical images of insulin-loaded AX microcapsules during transit in the simulated stomach (**a**), small intestine (**b**), and ascending colon after 6 h (**c**) and 12 h exposure (**d**).

Figure 2 shows the optical images of the insulin-loaded AX microcapsules recovered from the simulated human G.I. fluids media representing different phases of digestion, after an exposure based on the estimated maximum retention time in the human G.I. tract. The tested AX microcapsules remained morphologically stable during the simulated gastric incubation (2 h, pH ≤ 2) (Figure 2a) but behaved differently in the subsequent simulated intestinal transit. In the simulated small intestine incubation period (4 h, pH 7.2–7.4), the insulin-loaded AX microcapsules maintained their spherical structure, with a slight increase of size due to swelling after 6 h of exposure to G.I. fluids (Figure 2b). These results revealed that the AX microcapsules were stable in SGF and SIF and were not affected by the action of pepsin and pancreatin. Figure 2c,d shows the morphology of insulin-loaded AX microcapsules after 6 h and 12 h exposure in ascending colon fluid, respectively. After six hours, the morphology of the microcapsule was observed with a fragile eroded surface, which then broke up with the time of exposure (Figure 2d). These results agree with those reported in a previous study [10], where scanning electron microscopy images revealed that the AX gel structure collapsed after 18 h, due to bacterial degradation. The microcapsules were mainly broken down in ascending colon, where about 70% of microcapsules lost their structural integrity after 18 h in the simulated colonic fluid for the ascending colon (CA). Moreover, the structure of control samples, which were immersed in s-SCF sterile CA fluid after 12 h, remained constant, and no disintegration was observed. Therefore, these results showed that the breakdown of AX microcapsules was associated with a microflora-activated mechanism.

### 2.3. In Vitro Insulin Release

**Figure 3:**
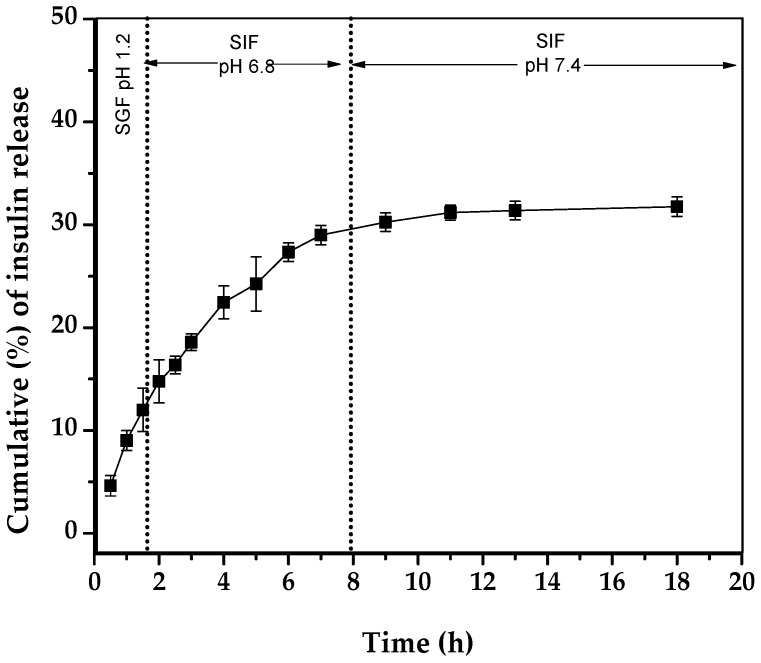
In vitro release profile of insulin from AX microcapsules incubated in simulated gastric fluid (SGF, 2 h) and simulated intestinal fluid (SIF, 6 and 10 h). Data represent the mean ± SD, *n* = 3.

The insulin release profile of the AX microcapsules was examined in a pH range of 1.2–7.4, to reflect GI pH conditions (Figure 3). In SGF, AX microcapsules released about 11% of their insulin content in 2 h. When the microcapsules were transferred to SIF (pH 6.8 and 7.4), the insulin was gradually released within the first 8 h of the studied period. At the end of the experiment, the total amount of insulin released from AX microcapsules was about 31%. The low insulin release values obtained may be associated with the covalent crosslinking of the AX microcapsule and its stability in SGF and SIF, as observed in Section 2.2, Figure 2. Therefore, the AX microcapsules minimized the insulin loss in the upper GI tract and retained a major part (70%) of insulin in their matrix, thereby facilitating reaching the colon and being released during AX microbial fermentation.

### 2.4. Effect of AX Microcapsules on the Simulated Gut Microbiota

**Figure 4:**
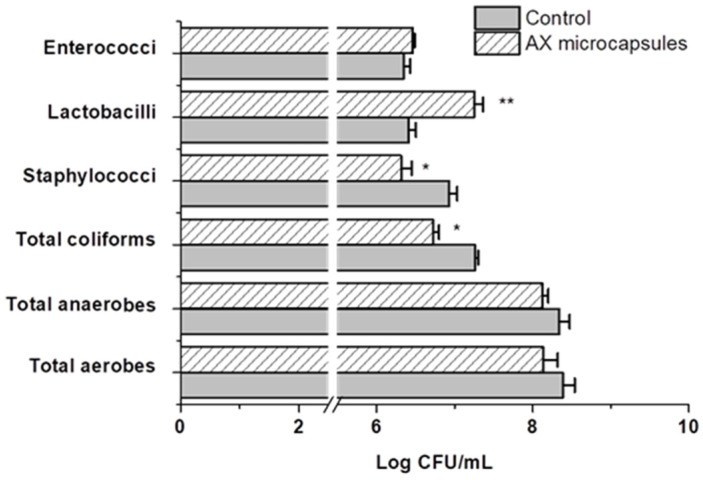
Average (±SEM) plate count measurements, expressed in log CFU mL^−1^, for different microbial groups untreated (control) and with treatment with AX gels microcapsules. Significant increases compared to the control are indicated by **, while significant decreases are indicated by * (*p* < 0.05) (*n* ≥ 4 per group).

Plate counts and quantitative PCR (RT-PCR) analysis were performed, to evaluate the effect of AX microcapsules administration on the microbial community composition. Figure 4 shows the plate count results for each period (average of all colon compartments). A plate count analysis for total aerobes and anaerobes in the different colon compartments delivered similar concentrations for both periods; the AX gels supplementation did not significantly change these levels. Looking at more specific groups, lactic acid-producing bacterial levels, such as *Lactobacilli*, registered a significant increase in both the ascending and descending colon compartments (*p* < 0.05). No significant changes were observed in the plate count of fecal coliforms and *staphylococci* groups between colon compartments, but compared with the control period, a significant reduction was observed. This reduction can be explained by a pathogen inhibitory effect associated with the increment of the lactic acid bacteria during the supplementation of arabinoxylans gels.

Table 1 provides an overview of the RT-PCR results for each colon compartment and different treatments. These results indicate that the addition of AX gels to the SHIME reactor resulted in a significant increase in the levels of *Bifidobacterium* in all colon compartments. In contrast, the *Lactobacilli* levels showed an increase only in the ascending colon compartment (*p* < 0.05); both bacterial group populations are considered to have beneficial health effects on humans. Some species of the *Bifidobacterium* group are regarded as excellent AX degraders [20,21]. Similar results were found in a study with diabetic rats, where the treatment with high-crosslinked AX microspheres restored levels of the genus *Lactobacillus* and *Bifidobacterium* [12].

Therefore, the loss of the structural integrity of microcapsules observed in the ascending colon compartment (Figure 2c,d) was correlated with the increase of *Bifidobacterium* levels in this region. In contrast, the abundance of genus *Bacteroides-Prevotella* decreased in the ascending and transverse colon compartments (*p* < 0.05). This bacterial group, mainly *Prevotella ssp*, has been related to mucin degradation and the development of insulin resistance [22,23]. Moreover, the levels of the family *Enterobacteriaceae* (*p* < 0.05) were reduced in the transverse and descending colon compared to the untreated control. This group of bacteria includes opportunistic and overt pathogens responsible for many infections. The positive effect associated with an increase in beneficial bacteria and a reduction of opportunistic pathogens is consistent with the findings of others in non-cross-linked arabinoxylans [21].

### 2.5. Metabolic Activity

**Figure 5:**
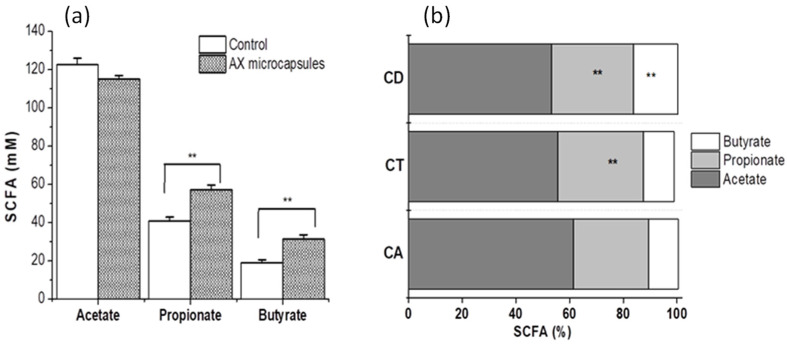
Average net SCFA production during control and AX microcapsule treatment (**a**). The average percentages of SCFA production in different colon compartments (CA, ascending; CT, transverse, and CD descending) of the SHIME system during treatment with AX microcapsules (**b**). Data represent the mean ± SD, *n* ≥ 4 per group. ** *p* < 0.05 compared to control.

Short-chain fatty acid (SCFA) concentrations were analyzed in different colon compartments during AX microcapsule fermentation, and means were calculated for the last 4 days of the treatment period and compared with the control period (Figure 5). Supplementation of AX microcapsules gave an overall increase of around 12% in SCFA production (*p* < 0.05) (Figure 5a). This increase of SCFA level upon cross-linked AX treatment is consistent with findings previously reported [10,24]. The total SCFA concentration (206.4 mM, data not shown) measured in the AX microcapsules treatment was divided into 59% acetate, 30% propionate, and 11% butyrate. The percentages of propionate increase significantly in the transverse and descending colon, while the production of butyrate in descending colon was the highest (Figure 5b). A higher propionate production by the breakdown of AX in the more distal colon has previously been described [25]. The decrease of butyrate in the ascending and transverse colon was in good agreement with the reduction in Bacteroides/Prevotella genera abundance (1–2 log CFU, Table 1) in this region, which are typical butyrate producers [26]. The production of SCFA is considered beneficial for the host because, among other benefits, it stimulates colonic blood flow, protects the host against pathogens, improves insulin sensitivity, and reduces cholesterol synthesis [27,28].

### 2.6. Hypoglycemic Effect and In Vivo Bioavailability of Insulin

**Figure 6:**
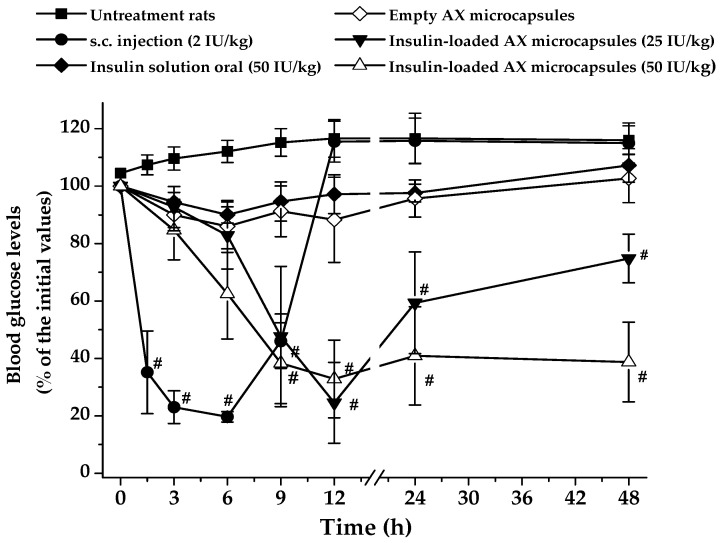
Hypoglycemic effect of various insulin formulations after oral or subcutaneous administrations to diabetic rats. Data represent the mean ± SD, *n* ≥ 5 per group. **#** *p* <0.05 compared to untreated rats, empty AX microcapsules, or oral insulin solution.

The pharmacological effect of insulin-loaded AX microcapsules was evaluated in diabetic rats dosed orally with 25 and 50 IU/kg. Changes in plasma glucose levels of rats were compared to rats dosed subcutaneously with free insulin at 2 UI/kg, rats receiving orally free insulin at 50 IU/kg, and rats receiving empty AX microcapsules (Figure 6). As expected, after subcutaneous insulin administration, blood glucose levels started to decrease significantly within the first 1 h. The maximum decrease of blood glucose level was about 80%, observed 6 h after injection, after which glycemia slowly increased, with starting hyperglycemic values achieved within 12 h post-administration. Outstandingly, the duration of the hypoglycemic effect was significantly prolonged after oral administration of both doses (25 and 50 IU/kg) of insulin-loaded AX microcapsules, with a significant difference between doses (*p* < 0.05) only after 48 h. The hypoglycemic effect appeared more pronounced after 6 h for the 25 IU/kg dose, but a faster onset was observed for a 50 IU/kg dose. The maximal reduction obtained in the blood glucose level was about 70% of its initial value, after 12 h for both doses. The most interesting point in the hypoglycemic effect profile was that normal glucose levels were maintained for about 48 h, which implied that a stable release with an appropriate amount of intact insulin occurred from 9 to 48 h. Given that non-encapsulated insulin did not affect blood glucose levels after oral administration, this suggests that AX microcapsules protect insulin in the gastrointestinal tract and allow its absorption in an active form. Thus, the hypoglycemic response of insulin ensnared in AX microcapsules is associated with the serum concentration of human insulin absorbed through the intestinal tract. Although, the empty AX microcapsules showed a slight hypoglycemic effect between 6 and 12 h after administration. This interesting tendency can be explained by the capacity of this polysaccharide or its gel to reduce postprandial glucose levels, as has previously been reported in diabetic rats [29,30].

Table 2 shows pharmacodynamic parameters closely related to pharmacological responses. The main finding is that oral administration of insulin-loaded AX microcapsules lowered the blood glucose levels (C_min_) to almost the same values as s.c. injection of insulin. Moreover, AX microcapsules needed 6 h more to induce the maximum decrease in glycemia (T_max_) than animals treated with s.c. insulin. On the other hand, insulin-loaded at a dose of 50 IU/kg induced an approximately 1.5-fold increase in the cumulative hypoglycemic effect (AAC_0–48 h_) compared with the values for a dose of 25 IU/kg. Finally, the insulin encapsulated in AX carriers increased its PA (%) by about 15-fold compared with non-encapsulated insulin at the same dose. The results obtained for the insulin-loaded AX microcapsules were better than those obtained for other systems for oral insulin administration [28,29,30]. This could be associated with the low susceptibility to changes in pH and ionic strength reported for the covalent matrix of AX [31,32], which permitted a small amount of insulin to be released in the upper gastrointestinal tract in comparison to other systems.

**Figure 7:**
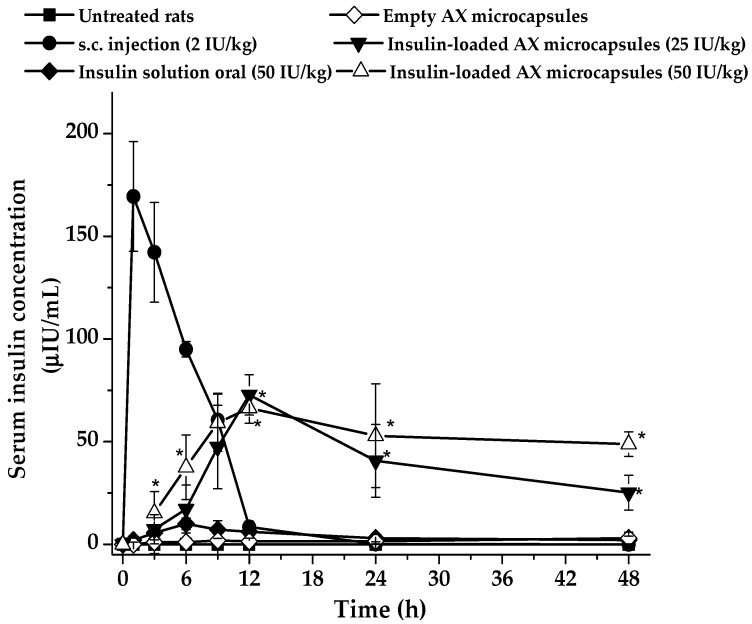
Serum human insulin levels of diabetic rats after oral or subcutaneous administration of formulations. Data represent the mean ± SD, *n* ≥ 5 per group. * *p* < 0.05 compared to s.c. injection.

The corresponding serum insulin concentrations after oral administration are shown in Figure 7. It was observed that the s.c. injection group resulted in a maximal insulin concentration at about 2 h after administration, indicating a direct and rapid absorption of insulin in the blood. This result is in line with previous reports [12,33]. When insulin-loaded AX microcapsules were administered orally, plasma human insulin levels also increased. Nevertheless, the peak value was lower, and the plasma insulin levels were maintained for 48 h, whatever the insulin dose in the AX microcapsule. However, the serum insulin profile showed that when the insulin dose increased from 25 to 50 IU/kg, the concentration of serum insulin after 24 h remained constant at that time. Both the maximal reduction of glucose levels and the increase of serum insulin corresponded to the time required for AX microcapsules to be degraded by the microbiota, according to the results of the in vitro tests carried out in the SHIME. These results confirmed that AX microcapsules appear to stabilize and protect entrapped insulin from degradation in the gastrointestinal tract and deliver it to the colon by a microbiota-active mechanism. However, the prolonged effect of the drug observed in AX treatments cannot be explained by the systemic insulin levels found; therefore, it could be related to the production of SCFA during AX degradation (Figure 5). These microbial metabolites have been reported to regulate glucose homeostasis by decreasing glucose production and increasing glucose uptake and glycogen synthesis in the liver [34].

Table 3 shows the pharmacokinetic parameters of the orally administered insulin-loaded AX microcapsules and the s.c. injection of 2 IU insulin/Kg derived from Figure 7. For the s.c injection, a typical profile characterized by a C_max_ reached during the first two hours post-administration, and a rapid descent during the following 6 h was observed. When insulin was orally administered with AX microspheres, whatever the insulin dose, the profile of the curve was characterized by a C_max_ reached 12 h post-administration, whose value was significantly lower than the C_max_ found for insulin s.c administered (*p* < 0.05). Interestingly, the elimination constant (K_el_) was found to decrease with AX microspheres formulation, indicating a slower rate of insulin elimination from the body, which was also obvious from the increased insulin half-life and consequently a prolonged drug release. This behavior may be related to a flip-flop pharmacokinetics, although further study is required to validate this effect. The relative oral bioavailability (FR%) of insulin encapsulated in AX microcapsules, in comparison with the subcutaneous route, was 13 for a dose of 25 IU/Kg and 8.7% for a dose of 50 IU/Kg. The serum insulin bioavailability of AX microcapsules was higher than values previously published with alginate/chitosan-based carriers (about 5%; [35]), lipid carriers (4.9%; [36]) or protein carriers (5.1% [37]). Thus, the results of this study clearly indicate that the encapsulation of insulin into arabinoxylans microcapsules allows the conservation of their biological activity when orally administered, with colon absorption and prolonged action in diabetic rats.

## 3. Materials and Methods

### 3.1. Materials

Arabinoxylans from maize bran were obtained and characterized as previously reported [38]. AX presented an A/X ratio of 0.72, Mw of 277 kDa, and an [η] of 2.68 dL/g. Laccase (benzenediol: oxygen oxidoreductase, E.C.1.10.3.2) from *Trametes versicolor*, human recombinant insulin (potency ≥ 27.5 U/mg), and all other chemical products were purchased from Sigma Chemical Co. (St. Louis, MO, USA).

### 3.2. Microencapsulation of Insulin

Free and AX-loaded insulin microcapsules were generated using a triaxial-electrospray (Spraybase^®^ system, Profector, Dublin, Ireland). The triple coaxial nozzle consisted of three stainless steel capillaries. The outermost capillary was connected to a power supply that was able to supply a high voltage through the conducting fluid to the innermost capillary. The solutions of insulin, AX, and laccase were prepared in agreement with those previously reported by Martinez-Lopez et al. [11]. These solutions were injected into capillaries using syringe pumps, at defined flow rates. In all experiments, the flow rates of the AX solution, insulin aqueous solution, and laccase solution were fixed to 4, 0.5, and 1 mL/min, respectively.

### 3.3. Characterization of Insulin-Loaded AX Microcapsules

The morphology and size of hydrated microcapsules were monitored by optical microscopic observations using an optical microscope (Olympus-BX51, Olympus American Inc. Center Valley, PA, USA). The particle size of insulin-loaded AX microcapsules was determined with a stage micrometer having an accuracy of 0.01 mm. The average sizes of 70 AX microcapsules were registered. The amount of insulin entrapped within polymeric nanoparticles was determined by high performances liquid chromatography [39].

### 3.4. Study of Insulin-Loaded AX Microcapsules in a Simulator of the Human Intestinal Microbial Ecosystem (SHIME)

#### 3.4.1. Simulation of the Human Gastrointestinal (G.I.) Tract

The human G.I. conditions used in this study were simulated in vitro using five sequential bioreactors. Each compartment imitates a different part of the human G.I. tract: the stomach and duodenum (reactor 1), small intestine (jejunum and ileum, reactor 2), ascending colon (reactor 3, CA), transverse colon (reactor 4, CT), and descending colon (reactor 5, CD) [15,27]. At the beginning of the experiment, the last three reactors (3, 4, and 5) were inoculated with bacteria from the fecal sample of three healthy adult volunteers (25-year-old male person), who had had no history of antibiotic treatment in the 12 months before fecal sample collection for the study. Inoculum preparation, retention time, pH, temperature settings, basal feed composition, and stabilization period were described previously [15]. The SHIME run consisted of a 2-week control period, during which basal feed medium was supplemented to the system, and a 3-week treatment period, during which AX gels powder was supplemented. During the treatment period, the starch (3 g) was replaced by an equal amount of AX gels powder in the feed for SHIME. It is important to note that the AX gels powder presented the same structural features as the AX microcapsules. The different evaluations of the insulin-loaded AX microcapsules were performed during the period of treatment with AX gels in the SHIME system.

#### 3.4.2. Morphology Changes of Insulin-Loaded AX Microcapsules in the Simulated Human G.I. Environment

To assess the resistance of the insulin-loaded AX microcapsules to the simulated human G.I. transit, they were exposed to the simulated human G.I. fluids, as described elsewhere [40]. At the same time, the insulin-loaded AX microspheres were exposed to 10 mL sterile simulated colonic fluid (s-SCF), as control for the biodegradation. The changes in the morphology of insulin-loaded AX microcapsules were observed under an inverted optical microscope, and microphotographs were taken using a digital camera (AmScope, FMA050). The observations of microcapsules were estimated in three randomly picked observation fields.

#### 3.4.3. Effect of AX Microcapsules on the Simulated Gut Microflora and Production of Metabolites

The bacterial populations were evaluated during the treatment period described above (Section 3.4.1), to investigate the influence of orally administered AX microcapsules on gut microflora. They were then compared with a control period (without AX microspheres). In both periods, bacterial populations were characterized using plate counts and quantitative PCR (RT-PCR). For the plate count, the bacterial groups were maintaned on specific media: BHI agar (total aerobes), BHI with cysteine (total aerobes, Oxford, Hampshire, UK), Rogosa agar (*Lactobacillus*), McConkey agar (coliforms, Difco), mannitol salt agar (*Staphylococci*, Oxoid), and Enterococcus agar (*Enterococci*, Difco). The samples were diluted serially in physiologic solution (8.5 g/L NaCl) and inoculated in three plates with a 0.1 mL sample of three dilutions and incubated at 37 °C (43 °C for coliforms), under aerobic or anaerobic conditions where necessary. Anaerobic incubation of plates was performed in jars with the gas atmosphere adjusted. The viable cell concentrations of the different microbial groups were determined as log10 CFU/mL, after proper incubations at the representative optimal conditions.

For RT-PCR, the samples were collected from SHIME on days 8, 11 13, 15, and 18 for the control period and on days 10, 14, 18, 21, and 24 for the treatment period. The samples (1 mL) were centrifuged for 2 min at 10,000× *g* at 4 °C, after which the supernatant was disposed of, and the pellet was suspended in 200 µL of physiological solution. DNA was extracted using a QIAamp DNA stool mini kit (Qiagen, Toronto, ON, Canada), following the manufacturer’s instructions. DNA concentration was determined by absorbance at 260 nm, and the purity was estimated using the A260/A280 ratio. RT-PCR for bacterial populations was performed as previously described [41], using the ROX RT-PCR Master Mix (2 ×) and specific RT-PCR primer sequences [42]. The template DNA in the reaction mixture was amplified (*n* = 2) and monitored with an Eco Real-Time PCR system (Illumina Inc., San Diego, CA, USA). The standard curves of individual RT-PCR assays were used for quantification of the target bacterial DNA from DNA samples of SHIME, as described previously [43]. Briefly, 6-fold dilution series of between 10 pg and 10 ng (ca 30–100 to 3 × 10^7^ target genomes) from the target species genomic DNA preparations were applied for PCR. The RT-PCR reaction specificity was determined by carrying out a melt curve analysis after amplification.

The microbial activity was monitored by its short-chain fatty acid (SCFA) production. Liquid samples (10 mL) from each reactor of the colon compartment were collected and frozen at −80 °C (the same days as the plate count sample). SCFA was performed as described previously [44]. The SCFA analysis was carried out using GC (Clarus 580, PerkinElmer, Waltham, MA USA) with a flame ionization detector and capillarity column (Elite-FFAP 30 m × 0.50 mm I.D; film thickness, 1 µm). Quantification of SCFA in samples was carried out using external calibration curves of acetic, propionic, and butyric acids, and the results were expressed as concentration (mM).

### 3.5. In Vitro Release of Insulin

The insulin release profile of the microcapsules was evaluated in simulated gastric fluid (SGF) (USP-NF XXV, pH 1.2) and simulated intestinal fluid (SIF) (USP-NF XXV, pH 6.5 and 7.4). Microcapsules (equivalent to 0.42 mg/mL insulin) were dispersed in a release medium, without enzymes and incubated at 37 °C under stirring (100 rpm). At appropriate time intervals, 1 mL of the solution was removed for analysis and replaced with fresh medium. The level of insulin released from the AX microcapsules into the SGF and SIF was measured by RP-HPLC, as described previously [11]. All the samples were analyzed in triplicate.

### 3.6. Study of Insulin-Loaded AX Microcapsules in Diabetic Rats

#### 3.6.1. Animals

Male Wistar rats were housed individually in stainless steel mesh cages under a controlled environment (room temperature: 23 ± 2 °C, relative humidity: 60 ± 5%, 12 h day/night cycle). A standard pellet diet and water were provided ad libitum during acclimatization. The animal ethical committee approved all the animal experiments, Research Center for Food and Development, CIAD, A.C., following the guidelines of the committee for control and supervision of experiments on animals (NOM-062-ZOO-1999), Government of Mexico.

#### 3.6.2. Hypoglycemic Effect and Bioavailability

Male Wistar rats of 250–300 g weight were fasted for 15- 19 h before inducing diabetes mellitus with free access to water. A solution of streptozotocin (STZ) at 20 mg/mL in citrate buffer at pH 4.5 was freshly prepared and used within 1 h. Before injection, a drop of blood was collected from the tail vein to determine baseline blood glucose levels. Each rat was injected with a single intraperitoneal dose of STZ solution (46 mg/kg), and hyperglycemia was induced about 3 days later. During the first 24 h, rats were given 5% glucose, to prevent hypoglycemia. Rats with fasting blood glucose levels higher than 300 mg/dL were randomly allocated into six groups of 6 rats each. The insulin-loaded microcapsules were administrated orally at 25, 50 UI/kg using an oral gavage needle. Positive controls received subcutaneous (s.c.) injection of 2 IU insulin/kg or oral administrations of insulin solution at 50 UI/kg. Negative controls received oral administration of empty AX microcapsules or were untreated. Blood was collected from the tail vein immediately before treatment and 3, 6, 9, 12, 24, and 48 h after administration. Blood glucose levels were measured using an Accu-Check Active blood glucose meter (Roche, Mannheim, Germany). Post-treatment blood glucose levels were expressed as the percentage of pre-treatment blood glucose. For quantitative serum insulin determination, the blood samples were centrifuged at 5000 rpm at 4 °C for 10 min, and the resulting serum was stored at −20 °C before analysis. The level of insulin in serum was then measured using a commercially available human insulin ELISA kit (Invitrogen, Life technologies™).

From the plasma glucose curve, the hypoglycemic effect was determined by calculating the area above the curve (AAC_0–48 h_) using the linear trapezoidal method. Pharmacological availability (PA) was estimated as the percentage of AAC of oral formulation compared to subcutaneous injection. The main pharmacokinetic parameters (C_max_, T_max_, and area under the curve (AUC_0–48 h_) were calculated from the representation of the serum insulin levels vs. time, using the PKsolver software, pKSolver®, a solver add-in for Excel® (Microsoft, Redmond, WA, USA) [45]. The relative oral bioavailability (Fr, expressed in percentage) of the oral insulin-loaded AX microcapsules against the subcutaneous administration of insulin was estimated as the ratio between the areas under the curve for the oral (AUC_oral_) and subcutaneous (AUC_sc_) administration.

### 3.7. Statistical Analysis

The means and standard error for all values were calculated. Standard errors were expressed at 1 to 2 significant figures and means at corresponding decimals of precision. Significance was determined by analysis of one-way analysis of variance (ANOVA) (OriginPro software, version 8.6. Originlab corporation, Northampton, MA, USA), followed by a Tukey’s multiple comparison test, and considered significant when *p* < 0.05.

## 4. Conclusions

The present study demonstrated that the breakdown of AX microcapsules is associated with a microflora-activated mechanism. In addition, the oral administration of AX microcapsules significantly increased beneficial populations and favorably modulated the microbiota composition in distal colonic compartments of a well-established dynamic human G.I. model. Furthermore, the results found with this in vitro model showed that AX microcapsules could resist the simulated conditions of the upper gastrointestinal system and be a carrier of insulin delivery to the colon. AX microcapsules lowered the serum glucose levels of STZ-induced diabetics by up to 70% of their basal glucose level. The hypoglycemic effect was observed for more than 48 h, and the relative oral bioavailability of insulin was around 13%. Therefore, this covalent AX microcapsule might serve as a promising alternative for insulin delivery to the colon, mediated by a microflora-activated mechanism.

## 5. Patents

There is a patent and a patent application resulting from the work reported in this manuscript.

## Figures and Tables

**Table 1:** Average (±SEM) abundance (log CFU mL^−1^) of microbial groups in SHIME compartments during untreated (control) and treatment with AX microcapsules.

Bacterial Group	Group	SHIME Compartment		
		Ascending	Transverse	Descending
All bacteria	Control	8.6 ± 0.3	7.8 ± 0.5	8.0 ± 0.6
	Treatment	8.8 ± 0.3	8.5 ± 0.3	8.5 ± 0.2
Genus Lactobacillus	Control	6.0 ± 0.1	6.4 ± 0.05	6.3 ± 0.05
	Treatment	6.9 ± 0.2 *	6.6 ± 0.1	6.3 ± 0.3
Genus Bifidobacterium	Control	5.6 ± 0.2	5.5 ± 0.3	5.6 ± 0.2
	Treatment	6.4 ± 0.2*	6.6 ± 0.2 *	6.9 ± 0.2 *
Phylum Bacteroidetes	Control	7.6 ± 0.2	7.3 ± 0.1	6.8 ± 0.4
	Treatment	7.7 ± 0.1	7.5 ± 0.2	7.3 ± 0.1
Genus Bacteroides/Prevotella	Control	9.2 ± 0.3	9.1 ± 0.3	8.5 ± 0.4
	Treatment	7.4 ± 0.04 *	8.2 ± 0.2 *	7.9 ± 0.2
Family Enterobacteriaceae	Control	7.9 ± 0.3	8.4 ± 0.3	8.6 ± 0.3
	Treatment	7.6 ± 0.2	7.3 ± 0.2 *	7.3 ± 0.1 *

Data represent the mean ± SEM, *n* ≥ 4 per group. * *p* < 0.05 compared to their respective control.

**Table 2:** Pharmacodynamic parameters after oral administration of insulin-loaded AX microcapsules in 25 and 50 IU/Kg or insulin solution (50 IU/Kg).

Insulin Formulation	Dose (IU/Kg)	AAC_(0–48 h)_ (% h)	T_max_ (h)	C_min_ (% Basal Glucose)	PA (%)
AX microcapsules	25	1800 ± 260	12	27.2 ± 9	19.9 ± 1.7 ***
AX microcapsules	50	2700 ± 320	12	32.5 ± 7	14.2 ± 1.2 ***
Oral insulin	50	190 ± 90	6	89.7 ± 3	0.9 ± 0.4

AAC: area above curve; T_max_: time at maximum effect was achieved; C_min:_ minimum relative basal glucose concentration in the blood; PA corresponds to the pharmacological activity. Data represent the mean ± SD at 1 to 2 significant figures, *n* ≥ 5 per group. *** *p* < 0.001 compared to oral insulin solution.

**Table 3:** Pharmacokinetics parameters for serum insulin levels in the diabetic rat model.

Insulin Formulation	Dose (IU/Kg)	C_max_ (µIU/mL)	T_max_ (h)	AUC_0–48 h_ (µIU*h/mL)	K_el_ (h^−1^)	t_1/2_ (h)	FR (%)
s.c. injection	2	178 ± 27	2	1197 ± 190	0.40 ± 0.041	1.7 ± 0.1	100
AX microcapsules	25	78 ± 19	12 *	1897 ± 390 *	0.052 ±0.008	13 ± 2	13 ± 1
AX microcapsules	50	73 ± 25	12 *	2597 ± 650 *	0.027 ±0.007	25 ± 8	8.7 ± 0.9

C_max_: maximum plasma concentration; T_max_: time at C_max_; K_el_: elimination rate constant; t_1/2_: half-life; FR: relative bioavailability. The data were obtained from Figure 5, and each value represents the mean ± SD at 1 to 2 significant figures (*n* = 5). * *p* > 0.05 compared to s.c injection.

## Data Availability

Data is contained within the article.
